# The Influence of a Microstructural Conformation of Oriented Floating Films of Semiconducting Polymers on Organic Device Performance

**DOI:** 10.3390/polym16050710

**Published:** 2024-03-05

**Authors:** Shubham Sharma, Kumar Vivek Gaurav, Shuichi Nagamatsu, Shyam S. Pandey

**Affiliations:** 1 Graduate School of Life Science and Systems Engineering, Kyushu Institute of Technology, 2-4 Hibikino, Wakamatsu, Kitakyushu 808-0196, Japan; kvgaurav2010@gmail.com; 2 Department of Computer Science and Electronics, Kyushu Institute of Technology, 680-4 Kawazu, Iizuka 820-8502, Japan; nagamatsu@phys.kyutech.ac.jp

**Keywords:** unidirectional floating film transfer method, semiconducting polymers, organic bistable memristor, organic field effect transistor, organic Schottky diode

## Abstract

Extended π-conjugation with backbone-planarity-driven π-π stacking dominates charge transport in semiconducting polymers (SCPs). The roles of SCP film morphology and macromolecular conformation concerning the substrate in influencing charge transport and its impact on device performance have been a subject of extensive debate. Face-on SCPs promote out-of-plane charge transport primarily through π-π stacking, with conjugated polymeric chains assisting transport in connecting crystalline domains, whereas edge-on SCPs promote in-plane charge transport primarily through conjugation and π-π stacking. In this work, we fabricated three different types of devices, namely, organic field effect transistors, organic Schottky diodes, and organic bistable memristors, as representatives of planar and vertical devices. We demonstrate that a planar device, i.e., an organic field effect transistor, performs well in an edge-on conformation exhibiting a field-effect mobility of 0.12 cm^2^V^−1^s^−1^ and on/off ratio >10^4^, whereas vertical devices, i.e., organic Schottky diodes and organic memristors, perform well in a face-on conformation, exhibiting exceptionally high on/off ratios of ~10^7^ and 10^6^, respectively.

## 1. Introduction

**Figure 1:**
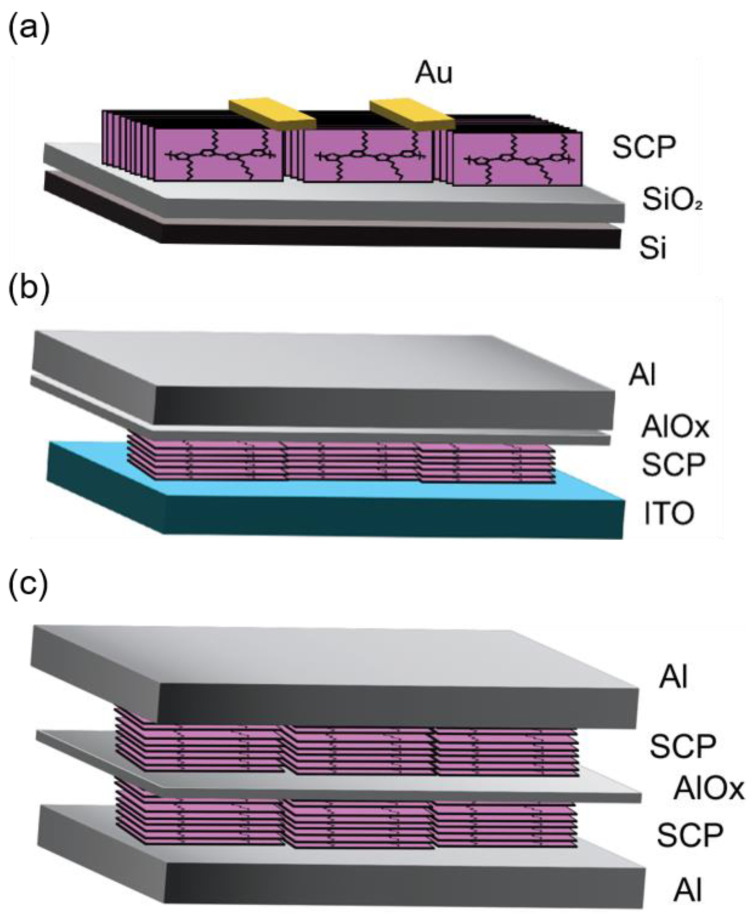
Device architecture for (**a**) OFET, (**b**) OSD, and (**c**) organic bistable memristor.

Semiconducting polymers (SCPs) have established themselves as outstanding organic semiconductors for widespread utilization in the rapidly growing area of plastic electronics. Because of their intrinsic molecular self-assembly propensity, SCPs’ optoelectronic characteristics can be easily adjusted [1,2,3,4,5]. SCPs have good solution rheology, making them appealing for use as an active layer of semiconductor devices in roll-to-roll manufacturing for flexible electronics. The rapid movement of charge carriers along the conjugation direction, subsequent interchain transport along the π stacking order, and ultimately inter-domain hopping collectively govern charge carrier transport in SCPs [6,7,8]. Understanding the relationship between polymer microstructure and charge transport mechanics is therefore critical for designing high-performance SCPs. A balanced optimization of intrachain and interchain charge percolation pathways is needed, which may involve tuning side chain design, molecular weight, and processing conditions. Further in-depth studies combining structural, optical, and charge transport characterizations of well-defined SCP systems are still needed to establish clear structure–function guidelines [9,10]. It is well acknowledged in the material engineering area that the transport of the charge carriers in SCPs is dependent on film morphology and structural arrangement [11,12,13]. Enhancing the ordering of the crystalline SCP films leads to heightened field-effect mobility (μ_FE_) when applied to planar or lateral configurations like those of organic field-effect transistors (OFETs). Simultaneously, it facilitates enhanced vertical or out-of-plane carrier flow in vertical configurations, exemplified by organic memristors as well as organic Schottky diodes (OSDs) and organic bistable memristors [11,12,13,14,15,16]. Furthermore, given the device architecture under consideration, the macromolecular conformation of the SCPs pertaining to the substrate plane is critical for effective charge transfer in the direction of the electrode [17]. In broad terms, SCP films have three sorts of macromolecular conformations: face-on, edge-on, and end-on [17,18]. According to a device’s design, endeavors have been made to optimize device performance by refining the conformation of SCPs in thin films. Given the significance of both the conjugation direction and π-stacking ordering pertaining to the substrate plane, the edge-on conformation of SCP thin films emerges as a crucial factor for planar devices like OFETs. On the contrary, a face-on conformation in SCP films, with the π-stacking direction aligned to the plane of the substrate, promotes carrier hopping in the vertical direction. This particular configuration proves advantageous for vertical devices such as OSDs and organic memristors [18,19,20,21,22]. The majority of recent studies have concentrated on one of the device geometries, either planar or vertical. For a variety of reasons, the real effect of such conformations on both types of devices at the same time is quite limited. The end-on conformation of the polymeric backbone in CP thin films was also found to have better out-of-plane charge transport [22]. A comparative analysis investigating the performance of simultaneous planar and vertical device architectures based on the same active material could provide significant insights. This could elucidate intrinsic structure–function relationships, separating out effects simply due to device engineering. Fabricating planar and vertical transistors on the same substrate with the same batch of polymer semiconductor allows a more controlled experiment. Additionally, studying films with varied processing conditions and thicknesses could reveal the impact of morphology and molecular ordering on charge transport anisotropy. While significant progress has been made in the development of planar and vertical organic electronic devices, a comprehensive understanding of their respective morphologies and performance characteristics remains a crucial area of investigation. Most prior works focused on only planar or vertical devices. More works systematically comparing both geometries are still needed to establish morphology–performance guidelines for optimizing both device types. The three types of devices discussed in this work for planar and vertical charge transport are OFETs (Figure 1a), OSDs (Figure 1b), and organic bistable memristors (Figure 1c). Here, the OFET is a planar device, whereas the OSD and organic bistable memristor are vertical devices. Comparing performance metrics across this combination of planar and vertical devices offers greater insight into the morphology–charge transport interplay along different orientations. The OFET, OSD, and memristor therefore represent complementary tools spanning planar and vertical configurations for relating SCP structure, orientation, and crystallinity to directional charge transport behavior.

**Figure 2:**
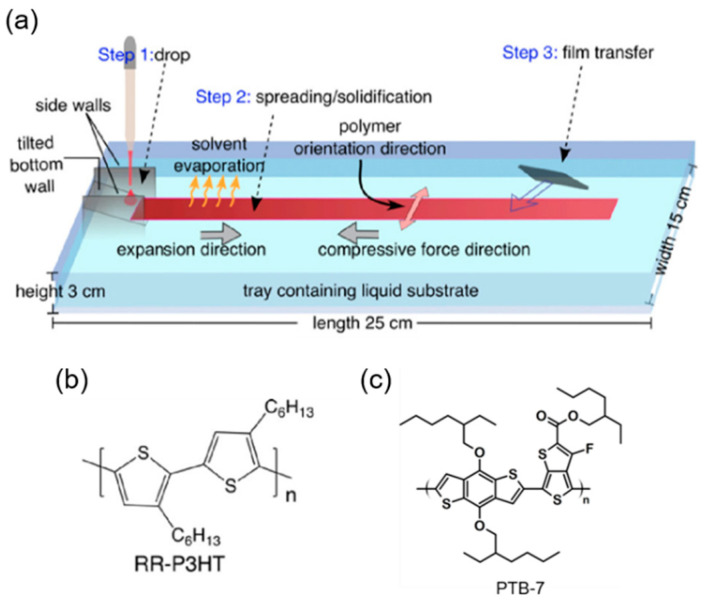
(**a**) Schematic diagram of UFTM and chemical structure of SCPs; (**b**) RR-P3HT; and (**c**) PTB-7.

While the conformation of SCPs may be regulated by careful molecular engineering, altering their molecular structure influences the crystallinity of the thin films, affecting their physical characteristics [19,23]. Additionally, due to the prevalence of the face-on molecular conformation, traditional methods such as friction transfer and solution shearing, involving the application of shear force to orient polymers, are not suited for producing planar devices such as OFETs [24]. Recently, our group discovered a simple methodology for producing thin films of organic SCPs: it is known as the unidirectional floating-film transfer method (UFTM) as shown in Figure 2a. In this method, a small amount of selected SCP ink (~8 μL) is dispensed into a hydrophilic orthogonal liquid pool, in which the SCP ink spontaneously spreads. During this process, the SCP ink solvent evaporates, forming a solid floating film that is subsequently cast to the target clean substrate. Concurrently, the viscous forces of the liquid substrate (LS), acting in the direction opposite to the film flow, impart a directional alignment to the floating film. This method allows for the production of oriented homogeneous films on an orthogonal LS, with the option to transfer them to a desired substrate [25]. Syafutra et al. successfully demonstrated the homogeneity of the orientations of UFTM films across different thicknesses [26]. Because of these benefits, we can utilize UFTM films for any device architecture. Furthermore, in this study, we demonstrate orientation control in the UFTM by adjusting the rheological parameters, which aids in the development of a stable system for thin-film formation. In a previous work, we reported the creation of multilayer thin films without affecting the underlying layers, constituting one of the most fascinating difficulties for most thin-film manufacturing processes, particularly solution processing.

SCPs exhibiting a greater density of hydrophobic side chains displayed an edge-on conformation after fabrication using the UFTM, rendering them well suited for planar device configurations. Conversely, SCPs featuring a lower density of hydrophobic side chains were anticipated to adopt a face-on conformation on the LS, thereby enhancing vertical charge transport. This strategic amalgamation of the UFTM with the judicious selection of SCPs and a LS holds promise in terms of achieving a desired backbone conformation, ultimately leading to optimal device performance. This systematic approach underscores the significance of tailoring material characteristics to specific applications in the realm of organic electronics [12,13,27,28,29]. In this work, we used two different types of polymers, RR-P3HT (Figure 2b) and PTB-7 (Figure 2c), and two different techniques of film fabrication, the UFTM and spin coating. We demonstrate that RR-P3HT assumes an edge-on conformation whereas PTB-7 assumes a face-on conformation when coated using the UFTM. Further, we also demonstrate that spin-coated thin films are dominantly face-on/mixed oriented. Three types of devices, OFET, OSD, and organic bistable memristors, are fabricated, and it is shown that the planar devices exhibit a preference for the edge-on conformation while vertical or sandwich devices favor the face-on conformation for their respective device performances.

## 2. Materials and Methods

### 2.1. Materials

High-quality RR-P3HT and octadecyl(trichloro)silane (OTS), along with super-dehydrated solvents (chloroform and toluene), were obtained from Sigma-Aldrich, Tokyo, Japan and used in their as-received states. PTB-7 was obtained from 1-molecule. LS (ethylene glycol and glycerol) dehydrated chloroform for making the SCP ink were purchased from Sigma Aldrich. Other solvents, like methanol, hexane, acetone, and isopropyl alcohol, used for washing the substrates, were also purchased from Sigma Aldrich.

### 2.2. Thin Film Characterization

Polarized UV-visible absorption spectroscopy: Electronic absorption spectra of the thin films deposited on glass substrates were recorded using a UV-visible spectrophotometer (JASCO V-570, Tokyo, Japan). For polarized absorption spectrum measurements, a Glan–Thompson prism was strategically positioned between the sample and the incident light source. 

Atomic Force Microscopy (AFM): AFM images of the films were captured using a scanning probe microscope (JSPM, Shimadzu, Tokyo, Japan) operating in contact mode and equipped with a silicon tip. Thin films cast on glass substrates were employed for this measurement.

X-ray diffraction (XRD): Si substrates were utilized for XRD and grazing-incidence X-ray diffraction (GIXD) measurements. The substrates underwent meticulous cleaning processes in acetone, isopropanol, and hexane ultrasonic baths, each lasting 10 min, to ensure optimal conditions for subsequent analyses. The films were characterized using out-of-plane X-ray diffraction (θ−2θ scan) conducted using a Rigaku X-ray diffractometer (Tokyo, Japan) with a Cu-K radiation source. Incident X-rays with a refractive index < 1 undergo total external reflection when their grazing incidence angle (ω) with respect to a film surface is <the critical angle (ωc). For in-plane grazing-incidence X-ray diffraction (GIXD) measurements (φ−2θχ scan), the sample and detector were rotated by angles of φ and 2θχ, respectively, while the scattering angles were set to 0.14° and 0.28° from the sample surface, respectively. Additionally, the angle (χ) between film propagation during UFTM and the scattering vector was fixed at 0° or 90° to investigate anisotropy in the thin film’s macromolecular architecture. For out-of-plane XRD measurements, the X-ray source and detector were rotated by angles of θ and 2θ, respectively, from the specimen plane. This comprehensive approach ensured precise characterization of the thin film’s structural features.

### 2.3. OFET Fabrication

OFETs were fabricated on highly doped p-type silicon substrates with a 300 nm thermally produced SiO_2_ dielectric possessing an intrinsic capacitance (C_i_) of 10 nFcm^−2^. The substrates underwent a thorough cleaning process to eliminate residual solvents through nitrogen blowing. Sequential washing steps included hydrophilic treatment of concentrated ammonium solution/H_2_O_2_/DI water = 1:1:50, followed by treatment of concentrated hydrochloric acid/H_2_O_2_/DI water = 1:1:50 solutions. Subsequently, the Si/SiO_2_ substrates were immersed in OTS solution in chloroform (20 mM) for 24 h to generate self-assembled monolayers (SAMs) on the Si/SiO_2_ substrates. Following SAM development, the substrates underwent additional ultrasonic cleaning in chloroform for 10 min and annealed at 200 °C for 30 min. UFTM thin films of RR-P3HT in chloroform (20 mg/mL) were produced in a 3:1 ratio of ethylene glycol/glycerol at 50 °C and cast onto the substrates. These films were then annealed at 100 °C for 30 min in a nitrogen glove box. Subsequently, gold-based source/drain electrodes were thermally evaporated (under 10^−6^ Torr) using nickel masks. The channel lengths (L) and widths (W) of the OFETs were 20 µm and 2 mm, respectively. The output and transfer characteristics of OFETs were obtained using a two-channel source-measure unit (Keithley 2612, Cleveland, OH, USA). This comprehensive fabrication process ensured precise control over the device parameters and reliable measurements for subsequent analysis.

### 2.4. OSD Fabrication

For OSD fabrication, ITO-coated glass substrate was employed as the ohmic contact. Substrates were thoroughly cleaned in the same manner as indicated above. To make thin films via spin-coating, RR-P3HT was dissolved in chloroform (20 mg/mL). Spin-coated films were created via spinning at 1000 rpm for 10 s, followed by 2000 rpm for 50 s. These films underwent annealing at 100 °C for 10 min within a nitrogen glove box, with gradual cooling. The upper aluminum (Al) electrode was thermally evaporated in a high-vacuum environment (10^−6^ Torr) using shadow masks with a 2 mm width, thereby creating a device area of 4 mm^2^. Al was used to establish a Schottky barrier. An aluminum oxide (AlO_x_) interlayer was also introduced between the Al contact and the SCP film. To achieve this, 10 nm of Al was evaporated as discussed previously onto P3HT, followed by exposure to oxygen-prone conditions for 1 h to allow for AlO_x_ interlayer formation, as previously documented [30].

### 2.5. Organic Bistable Memristor Fabrication

For the fabrication of organic bistable memristor, Al bottom contact was evaporated thermally under high vacuum conditions (10^−6^ Torr) with the help of a 2 mm wide mask. The thin films were cast using multilayered UFTM, where a small amount (~8 μL) of PTB7 ink dissolved in chloroform (20 mg/mL) was dropped at ambient temperature on ethylene glycol as an LS to form a thin film. The thin films were washed in methanol to remove the excess LS and dried using a nitrogen gun. In this way, 3 layers of UFTM thin films were coated. They were then vacuum-dried for 2 h and annealed at 100 °C for 15 min in a nitrogen environment. The morphological properties of multilayered UFTM thin films have been thoroughly researched and reported on in prior work [31]. Next, under high-vacuum conditions (10^−6^ Torr), 10 nm of Al was coated subsequently using thermal evaporation [32]. In summary, the creation of island-like structures occurred as a consequence of both the minimal thickness of the thermally evaporated metal on the organic layer and the restricted in-plane movement of the thermally evaporated metallic clusters. This effect was accentuated by terminating the deposition before clusters merged to form a continuous layer. Additionally, the AlO_x_-islands, generated through thermal evaporation with an approximately 0.1 Å/s evaporation rate, exhibited a multilayered structure comprising aluminum clusters covered with a thin oxide layer. This may be attributed to oxygen remaining in the evaporator. The polymer layers above the Al-islands were subsequently coated with a series of multilayered UFTM films (3 layers), followed by subjection to slow drying in a nitrogen glove box and annealing at 100 °C for 30 min. Finally, top electrodes, orthogonally patterned to the bottom electrode using a 2 mm broad shadow mask, were deposited by thermally evaporating a 70 nm thick piece of aluminum under high-vacuum conditions (10^−6^ Torr). The electrical properties of these memory devices were evaluated in a vacuum environment (10^−3^ Torr) using a Keithley 2612 source measure unit, with a positive bias applied to the top electrode during the assessment of electrical characteristics.

## 3. Results and Discussion

### 3.1. Thin-Film Microstructure

**Figure 3:**
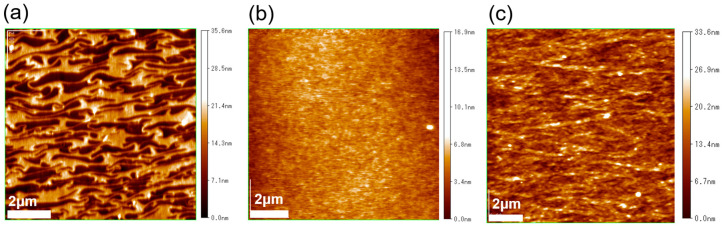
AFM images of (**a**) UFTM-coated RR-P3HT, (**b**) spin-coated RR-P3HT, and (**c**) UFTM-coated PTB-7.

The surface morphologies of the RR-P3HT and PTB-7 films, fabricated using the UFTM and spin-coating, were visualized using AFM measurements. The AFM images produced in tapping mode are shown in Figure 3. It can be observed that a distinct and unidirectional alignment of SCP domains in the RR-P3HT UFTM film is present. It was noted that these domains were perpendicular to the spreading of the film. In contrast, the spin-coated film in Figure 3b exhibits no evident characteristics, confirming its random and amorphous morphology. This observation underscores the effectiveness of the UFTM in inducing a specific orientation of SCP domains, contributing to the unique surface morphology of the resulting films. Finally, as shown in Figure 3c, the UFTM-treated PTB-7 thin film has polymer domains oriented parallel to the polymer’s orientation. As a result, the UFTM appears to have facilitated an anisotropic orientation with large-aligned domains in both the RR-P3HT and PTB-7 films. The RR-P3HT spin-coated films, on the other hand, exhibit a featureless surface morphology.

**Figure 4:**
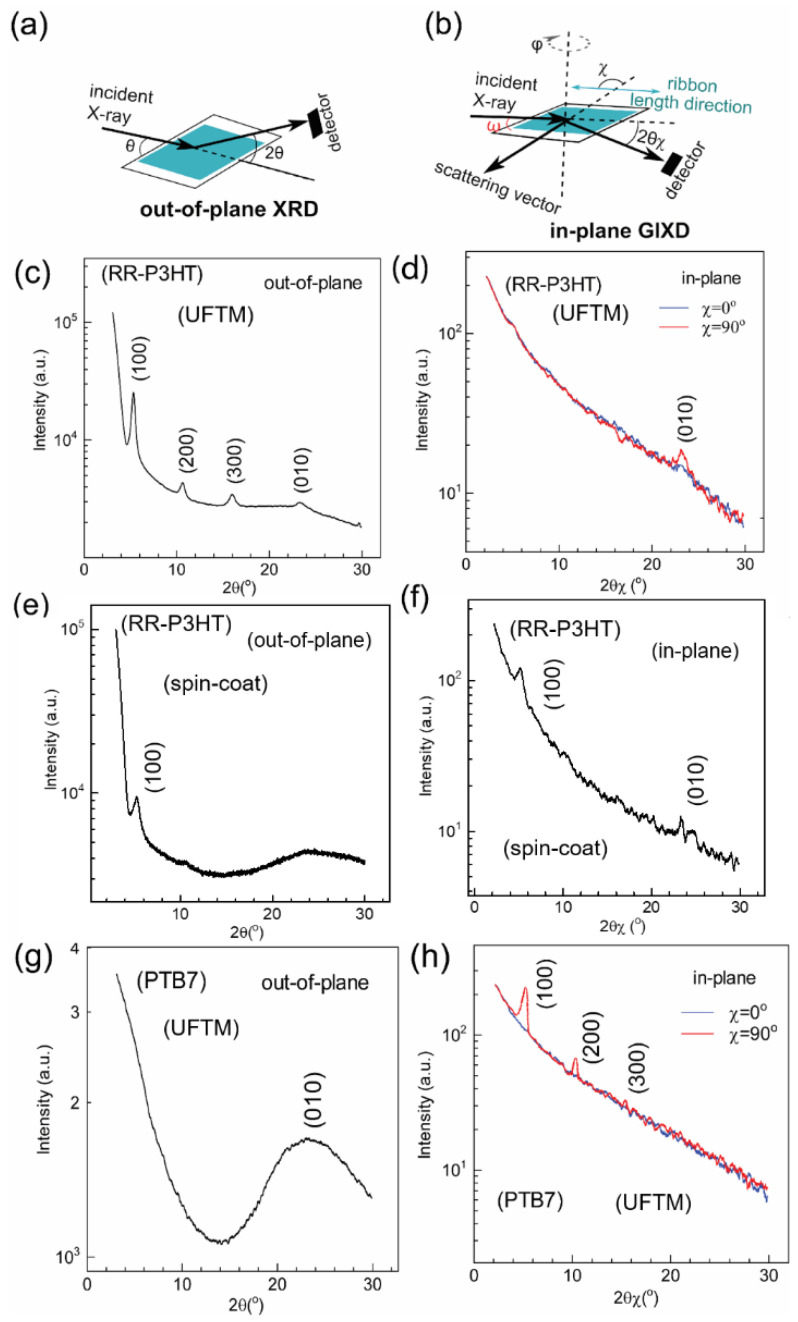
Schematic representation of (**a**) out-of-plane XRD and (**b**) in-plane GIXD measurements. (**c**,**e**,**g**) out-of-plane XRD profile and (**d**,**f**,**h**) in-plane GIXD profile for the UFTM-produced films of RR-P3HT, spin-coated films of RR-P3HT, and UFTM films of PTB-7, respectively. For the in-plane GIXD measurements, the scattering vector was kept parallel (χ ≈ 0°) or perpendicular (χ ≈ 90°) to the film length direction.

Subsequently, out-of-plane XRD and in-plane GIXD experiments were conducted to examine film crystallinity and visualize the macromolecular conformations, as shown in Figure 4 [33]. The UFTM RR P3HT films, as analyzed through out-of-plane XRD, displayed several strong diffraction peaks of (h00), which correspond to the lamellar alkyl stacking up to the third order (Figure 4c). In the in-plane GIXD, the UFTM films exhibited no diffraction peaks related to alkyl chains, and only a (0k0) diffraction peak associated with π-stacking was observed (Figure 4d). This suggests that all crystallites pertaining to the RR P3HT UFTM films are oriented in an edge-on fashion [34]. The UFTM thin films were also highly crystalline, as seen in the XRD patterns. In contrast, the spin-coated films exhibited only small (h00) diffraction peaks of the first order, indicating that the films produced via spin-coating were relatively amorphous. Notably, the in-plane GIXD for the spin-coated films displayed peaks of (100) and (010), suggesting the presence of crystallites in a mixed edge-on/face-on phase (Figure 4e,f) [34].

Finally, in the PTB-7 UFTM-coated thin film, the out-of-plane XRD results exhibited a clear (010) peak corresponding to π-π stacking, whereas there was an absence of (h00) peaks corresponding to alkyl chains. Further, in the in-plane GIXD measurement for the PTB-7 thin films, (h00) alkyl chain peaks up to the third order were present, and there were no (0k0) π-π stacking peaks (Figure 4g,h) [35]. This shows that the crystallites of PTB-7 are face-on-oriented. Upon observing the XRD related data, it is clear that the UFTM-coated thin films are edge-on orientated. This is because RR-P3HT contains hydrophobic alkyl chains that interact with the hydrophilic LS. The spin-coated RR-P3HT thin films, on the other hand, have a mixed (edge-on/face-on) conformation. Finally, the hydrophilic parts of the side chains of the UFTM-coated PTB-7 thin films established hydrogen bonds with the hydrophilic LS, resulting in a face-on conformation.

### 3.2. Device Characteristics

#### 3.2.1. OFET Performance

**Figure 5:**
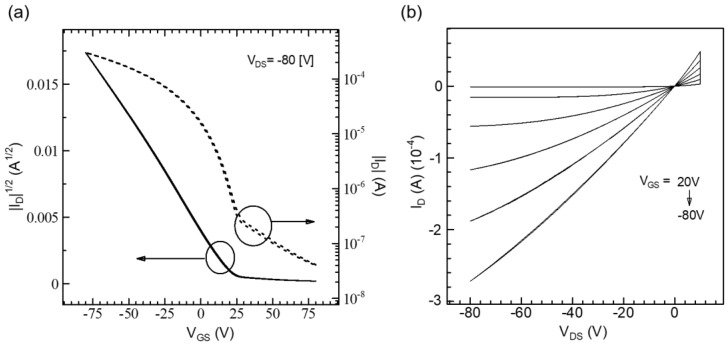
(**a**) Transfer (solid line for left-axis and dotted line for right-axis) and (**b**) output characteristics of UFTM-coated RR-P3HT OFETs.

The influence of macromolecular conformation was first verified in planar devices through the fabrication of OFETs. Figure 4 illustrates the transfer and output characteristics of OFETs for the RR-P3HT films produced using the UFTM. In the UFTM films, the alignment of the channel direction was maintained parallel to the orientation of the polymeric domains. The field effect μ was calculated using transfer curves in the saturation regions [36,37]. The saturated μ was estimated from the slope of the saturated |I_DS_|^1/2^—V_GS_ curve. The on/off ratio of the transfer characteristic was determined by calculating the ratio of on to off currents. The output characteristics of the OFETs (Figure 5a,b) clearly indicate a p-type feature. The μ measured from the saturation area was 0.12 cm^2^V^−1^s^−1^ (I_on_/I_off_ ~10^4^). The OFETs with a UFTM active layer films performed exceptionally well due to their improved crystallinity and edge-on orientation. This device performance for RR-P3HT thin films is higher than that observed for other techniques reported in the literature and is compared in Table 1 below [17].

#### 3.2.2. OSD Performance

**Figure 6:**
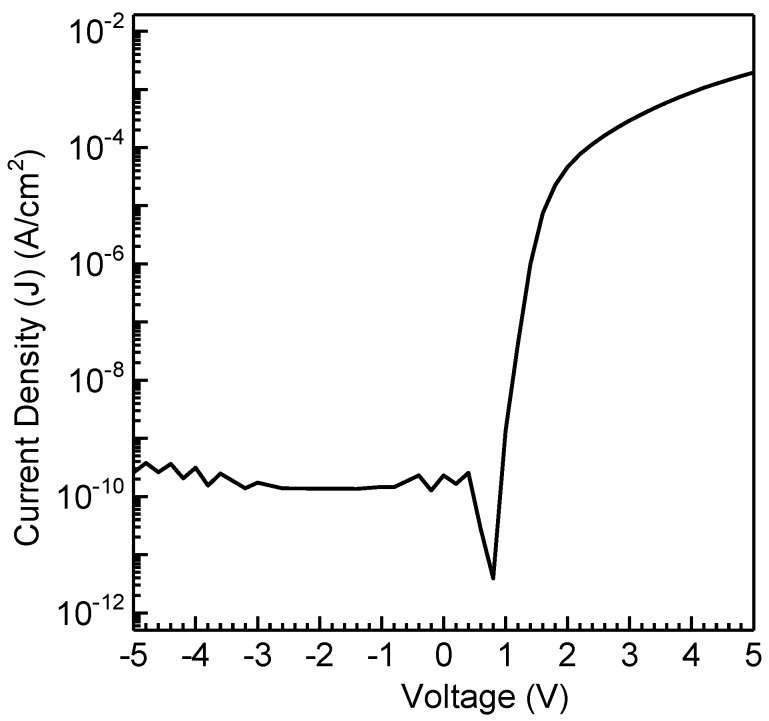
J−V characteristics of spin-coated RR-P3HT.

OSDs were developed by spin-coating RR-P3HT films exhibiting mixed face-on/edge-on orientations to explore the impact of molecular conformation on vertical charge transport. The resulting current density vs voltage (J−V) curves for the fabricated OSDs are depicted in Figure 6, showcasing distinctive asymmetric OSD features. This asymmetrical J−V profile distinctly illustrates that current flows with ease in one direction while encountering significant hindrance in the other. The establishment of a Schottky barrier at the AlO_x_/P3HT interface is attributed to the observed Schottky phenomenon. The thermionic emission model, as outlined in the reference for Schottky barrier diodes, has been widely accepted for its application to the organic analogue [38,39,40,41]. The rectification ratio (RR) was determined by the ratio of the forward to the reverse current at the same applied bias. Despite the observed high crystallinity, better macromolecular conformation, and anisotropic films achieved through the UFTM, spin-coated films exhibited significantly enhanced performance in OSDs compared to RR-P3HT. The spin-coated films of RR-P3HT exhibited an RR of 8.6 × 10^6^, which is exceptionally high compared to that of the UFTM films [34]. Furthermore, the ideality factor, which reflects deviations from ideal diode behavior due to non-idealities such as traps, recombination centers, and interface states, was estimated to be 2.32. This value is considered reasonable for organic devices. This highlights the pivotal role of molecular conformation in the regulation of vertical charge transfer in OSDs. 

#### 3.2.3. Organic Bistable Memristor

**Figure 7:**
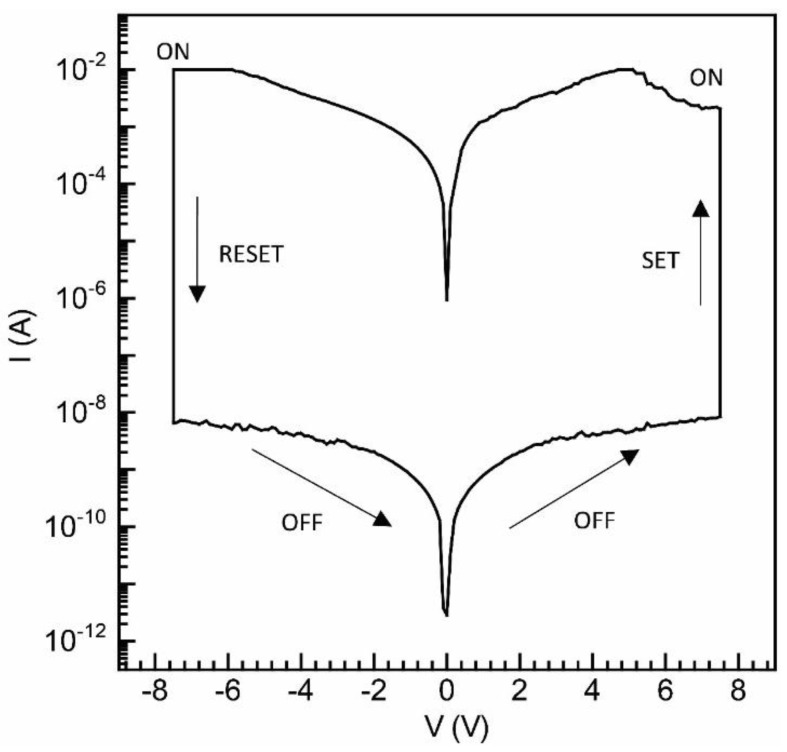
I−V plot for UFTM thin film using PTB-7-based resistive switching memory devices.

The current–voltage (I−V) characteristics of sandwich devices, created by employing a multilayered fabrication of PTB-7 films using the UFTM, are depicted in Figure 7. In this case, the memory devices displayed a sharp hysteresis loop, a frequently observed phenomenon in resistive switching memristors. This distinctive pinched hysteresis loop in the I−V characteristics signifies the resistive switching behavior characteristic of memory devices and is indicative of their potential utility in data storage applications [42]. This device demonstrates an on-current exceeding 10^−2^ A under both biases, as shown in the figure. It undergoes an external electrical bias sequence of 0 V/+7 V/0 V/−7 V/0 V, as indicated by the arrows. During the forward scan, a high resistance state (HRS), corresponding to the OFF state, is achieved. As the positive voltage increases, the current rises and abruptly shoots up at 6.4 V, signifying the transition from the HRS to the ON state, also known as the low resistance state (LRS). This process, known as the writing or SET state, involves transitioning from low to high current values. Notably, even after removing the bias, the LRS persists and refrains from reverting to the HRS. During the reverse sweep, the device remains turned on between 0 V and −7 V. A subsequent OFF state is observed at −7 V in the presence of the reverse bias voltage, resembling the ‘erasing’ procedure in memory devices. The devices can be reprogrammed, displaying a non-volatile reprogrammable behavior. Bisection occurs when two resistive states, i.e., an HRS and an LRS, are present. The retention of these states persists even after the removal of bias, validating the non-volatile memory effect. Reversible switching across the HRS and LRS repeatedly presents a potential for application in a rewritable data storage system. At V_read_ = 1 V, the currents for the LRS and HRS are 7.2 × 10^−3^ and 3.9 × 10^−9^ A, respectively, resulting in a current on/off ratio (I_on_/I_off_) of approximately 2.1 × 10^6^. PTB-7, with a dominating face-on conformation and crystalline domains, exhibiting superior pinched hysteresis loop characteristics [32,35,42,43].

Finally, Table 1 presents the recent trends in (a) OFETs, (b) OSDs, and (c) organic memristors, offering a comparative overview that includes the positioning of our devices within this context. We have compared RR-P3HT-based OFETs and OSDs with different techniques, while for organic memristors, various polymers and techniques recently used have been reported.

**Table 1:** Comparison table regarding recently reported (a) OFETs, (b) OSDs, (c) organic memristors.

**(a) OFETs:**			
**Technique**	**µ (cm^2^V^−1^s^−1^)**	**On/Off Ratio**	**Reference**
UFTM	0.12	10^4^	This work
Drop casting	8.4 × 10^−2^	10^4^	[44]
Solvent-drop casting	0.16	10^4^	[45]
Spin coating	3.8 × 10^−2^	2.4 × 10^5^	[46]
Spin coating	1.4 × 10^−2^	1 × 10^4^	[47]
Dip coating	8.5 × 10^−2^	1.5 × 10^4^	[48]
**(b) OSDs:**			
**Technique**	**On/Off Ratio**		**Reference**
Spin coating	8.6 × 10^6^		This work
Nanofiber solution casting	1.2 × 10^3^		[49]
Wet deposition	10^6^		[50]
Spin coating	1.11 × 10^6^		[30]
Spin coating	10^3^		[30]
Spin coating	8.8 × 10^2^		[38]
**(c) Organic memristors:**			
**Polymer**	**Technique**	**On/Off Ratio**	**Reference**
PTB-7	UFTM	2.1 × 10^6^	This work
P3HT:LiClO_4_	Spin coating	10^3^	[51]
PVA	Spin coating	10^4^	[52]
Cu-TCNQ	Vapor deposition	4 × 10^2^	[53]
PVA-PEDOT: PSS	Spin coating	10^2^	[54]

## 4. Conclusions

The UFTM and spin-coating processes were used to create large-area-orientated thin films of RR-P3HT and PTB-7. The polymers in the UFTM films coated with RR-P3HT were edge-on orientated, the spin-coated RR-P3HT films had mixed (face-on/edge-on) domains, and the UFTM films coated with PTB-7 were dominantly face-on oriented, according to film characterization employing X-ray diffraction. The macromolecular conformation of SCPs in thin films in relation to charge transport directions has a substantial influence on the performance of a device; thus, their role in device performance was investigated by fabricating planar (OFET with UFTM) and vertical (OSD and organic bistable memristor) devices with spin-coated and UFTM films, respectively. The RR-P3HT-based UFTM film with an edge-on conformation showed superior electrical performance in OFET devices (planar devices), RR-P3HT-based spin-coated films with an edge-on/face-on orientation (mixed orientation) exhibited excellent device performance in OSD devices (vertical devices), and PTB-7-based UFTM films with a completely face-on conformation exhibited excellent performance in organic bistable memristors (vertical devices).

## Data Availability

Data are contained within the article.
